# Dietary intake of potassium, vitamin E, and vitamin C emerges as the most significant predictors of cardiovascular disease risk in adults

**DOI:** 10.1097/MD.0000000000039180

**Published:** 2024-08-09

**Authors:** Yue Wang, Liyuan Han, Shiliang Ling, Yuyi Sha, Hongpeng Sun

**Affiliations:** a School of Public Health, Medical College of Soochow University, Suzhou, China; b Stomatology Hospital affiliated to Suzhou Vocational Health College, Suzhou, China; c Key Laboratory of Diagnosis and Treatment of Digestive System Tumors of Zhejiang Province, Ningbo No.2 Hospital, Ningbo, China; d Center for Cardiovascular and Cerebrovascular Epidemiology and Translational Medicine, Ningbo Institute of Life and Health Industry, University of Chinese Academy of Sciences, Ningbo, China; e Department of Oncology, Ningbo Hospital of Traditional Chinese Medicine, Ningbo, China; f Department of Rehabilitation Medicine, Ningbo No.2 Hospital, Ningbo, China.

**Keywords:** cardiovascular disease, machine learning, micronutrient, prediction model, UK Biobank

## Abstract

Prediction models were developed to assess the risk of cardiovascular disease (CVD) based on micronutrient intake, utilizing data from 90,167 UK Biobank participants. Four machine learning models were employed to predict CVD risk, with performance evaluation metrics including area under the receiver operating characteristic curve (AUC), accuracy, recall, specificity, and F1-score. The eXtreme Gradient Boosting (XGBoost) model was utilized to rank the importance of 11 micronutrients in cardiovascular health. Results indicated that vitamin E, calcium, vitamin C, and potassium intake were associated with a reduced risk of CVD. The XGBoost model demonstrated the highest performance with an AUC of 0.952, highlighting potassium, vitamin E, and vitamin C as key predictors of CVD risk. Subgroup analysis revealed a stronger correlation between calcium intake and CVD risk in older adults and those with higher BMI, while vitamin B6 intake showed a link to CVD risk in women. Overall, the XGBoost model emphasized the significance of potassium, vitamin E, and vitamin C intake as primary predictors of CVD risk in adults, with age, sex, and BMI potentially influencing the importance of micronutrient intake in predicting CVD risk.

## 1. Introduction

Cardiovascular disease (CVD) is a significant global health concern, contributing to both illness and death.^[1]^ Research has focused on micronutrients, such as vitamins and minerals, and their potential impact on CVD prevention. Recent studies have indicated that following a Mediterranean or vegetarian diet, which is high in micronutrients, may be linked to a reduced risk of CVD.^[2–4]^ Observational data has also suggested that adequate intake of iron, potassium, magnesium, vitamin E, vitamin C, vitamin B6, and folate could lower the risk of CVD.^[5–10]^ However, the precise relationship between micronutrients and their role in preventing or reducing the risk of CVD is still not fully understood. A systematic review from 2018 suggested that, apart from B vitamins, the intake of micronutrients may not significantly impact CVD risk.^[11]^

The role of individual micronutrients in maintaining cardiovascular health remains uncertain, as nutritional epidemiology studies have uncovered intricate relationships and interactions between nutrients and disease.^[5,12,13]^ Therefore, the simplistic linear models commonly employed in these studies may fail to consider potential interactions, resulting in conflicting findings.^[14]^

Machine learning methods, unlike traditional regression models, have the ability to detect and analyze intricate relationships between nutrient intake and CVD.^[14,15]^ Therefore, our objective was to develop a machine learning model using data from a substantial UK Biobank cohort to forecast the likelihood of CVD based on the consumption of various micronutrients, as well as to assess the significance of each nutrient in relation to this risk. This insight could hold clinical importance and enhance the strategies for preventing and treating CVD in the adult population.

## 2. Methods

### 2.1. Study and participants

The UK Biobank conducted a cohort study between 2006 and 2010, enrolling over half a million participants aged 40 to 69 from various regions of the UK. The study included an equal distribution of male and female participants who underwent a thorough assessment and completed a touchscreen questionnaire at the beginning of the study. More details about the UK Biobank can be accessed on their website at https://biobank.ndph.ox.ac.uk/. The Northwest Multi-Center Research Ethics Committee approved UK Biobank, and all the participants had provided written consent. Approval for this study was granted by the UK Biobank Resource under Application Number 78619.

A total of 126,456 UK Biobank participants who completed at least two online 24-hour dietary recall assessments were included in the study (N = 126,456). Exclusions were made for individuals with missing or incomplete data, as well as those who reported or were diagnosed with CVD or cancer at baseline, resulting in a final sample size of 90,167 participants. Follow-up was conducted from the recruitment date until the onset of CVD, death, or the study’s conclusion in 2021, whichever came first. The process of participant selection is presented in Figure S1, Supplemental Digital Content, http://links.lww.com/MD/N342

### 2.2. Assessment of micronutrient intake and other covariates

From 2009 to 2012, UK Biobank conducted a study where participants who provided their email addresses were asked to complete an online 24-hour dietary recall assessment every 3 to 4 months, with the goal of obtaining 5 assessments per participant.^[16]^ The majority of participants completed at least one online assessment during this time period, and mean intakes were calculated from the data collected. The assessments inquired whether participants had consumed any of 206 foods and 32 beverages in the previous 24 hours, and if so, the quantity of each item consumed. By considering the reported frequency of consumption, standard portion size, and nutrient content, the participants’ average daily nutrient intakes were determined. These results were compared to a 24-hour dietary recall completed with an interviewer on the same day, yielding a Spearman correlation coefficient of 0.6 (0.5–0.9) for most nutrients.^[17]^ The analysis included data on the intake of 11 micronutrients, with participants grouped into four quartiles based on their intake levels of these micronutrients.

Seven potential risk factors for CVD were assessed at baseline, including age, sex, body mass index (BMI), smoking status, drinking habits, systolic blood pressure (SBP) and Townsend deprivation index (TDI). Drinking habits were evaluated through a 24-hour alcohol intake assessment.

### 2.3. Outcome assessment

Disease outcomes were assessed using inpatient hospital and death registry data from the UK Biobank. The main focus of our study was on total CVD events, encompassing CVD-related mortality, coronary artery disease (CAD) and stroke. Additionally, individual CAD and stroke events were examined as secondary outcomes, including both fatal and non-fatal incidents. CVD-related deaths were identified using the International Classification of Diseases, 10th revision (ICD-10) codes I00–I99. CAD was defined by myocardial infarction (ICD-10 codes I21 and I22), heart failure (I11, I13, I25, I42, I50), and atrial fibrillation (I48). Stroke subtypes were categorized as hemorrhagic stroke (I60-I62), ischemic stroke (I63), unclassified stroke (I64), and transient ischemic attack (G45).

### 2.4. Data processing

The dataset was split into a training set (80% of the data) and a testing set (20% of the data). Given the imbalance between controls and cases in our dataset, which can lead to reduced algorithm performance, we addressed this by balancing the training set. We utilized the synthetic minority oversampling technique (SMOTE)^[18]^ to create synthetic samples of the minority class. This involved generating new instances that interpolate between existing minority class instances, considering the Euclidean distance between k-nearest neighbors,^[19]^ and linear combinations of features from existing minority-class neighbors.

To address discrepancies in micronutrient intake caused by variations in total energy intake among individuals, adjustments must be made during micronutrient assessments. The residual method^[20]^ was employed to adjust for total energy intake, where the residual intake is determined by subtracting the anticipated intake from the actual intake through a linear regression analysis of micronutrient intake in relation to total energy intake. Furthermore, we examined the linear relationships between the 11 micronutrients using Pearson correlation coefficients and illustrated the findings through heat maps depicting the correlations between each pair of features.

### 2.5. Modeling and statistical analysis

We utilized 5 machine learning algorithms, including eXtreme gradient boosting (XGBoost), random forests (RF), k-nearest neighbors (KNN), multilayer perceptron algorithm (MLP), and logistic regression, to develop predictive models for CVD risk based on nutrient intake data. The hyperparameters for XGBoost were optimized through manual grid search, varying the number of trees (100, 500, or 1000), learning rates (0.01, 0.1, 0.3), and tree depths (3, 4, 5). Similarly, the hyperparameters for RF were tuned by manual grid search, adjusting the number of trees, tree depths, “min_samples_leaf,” and “min_samples_split.” For KNN, the hyperparameters were fine-tuned via manual grid search, exploring the number of neighbors (3, 5, or 7), leaf sizes (10, 30, 50), and weight selection as “distance.” The hyperparameters for MLP were optimized through manual grid search, varying the hidden layer architecture (30, 50, 100), learning rates, and selecting “adam” as the solver. We employed GridSearchCV function to conduct 10-fold cross-validation for each set of parameters and identified the hyperparameter combination that yielded the best performance in cross-validation. Subsequently, we compared the performance of these prediction models based on metrics such as areas under the curve, accuracy, recall, specificity, and F1-score.

The study incorporated seven common predictors of CVD risk and intake data for 11 micronutrients. Furthermore, a separate model focusing solely on micronutrients was developed to investigate their direct influence on predicted CVD risk.

Three logistic regression models were developed for the study. Model 1 was constructed without adjusting for any confounders. Model 2 included adjustments for age (continuous) and sex (male or female). Lastly, Model 3 accounted for age (continuous), sex (male or female), ethnicity (white vs nonwhite), BMI categories (<25.0, 25.0–29.9, ≥30.0), income brackets (<18,000, 18,000–30,999, 31,000–51,999, ≥52,000), smoking status (never, previous, current), alcohol intake levels (g/d: nondrinker, 0–10, 10–20, ≥20), physical activity levels (min/day; low (<15), moderate (15–75), high (≥75)), years of education (0–7, 10–13, 15–20), and personal medical history of hypertension and diabetes (yes or no).

Subgroup analyses were conducted based on age (<65, ≥65 years old), sex (male or female), BMI (<30, ≥30.0 kg/m^2^), and smoking status (never or current) to investigate potential modifications to the relationship between micronutrient intake and CVD events. These risk factors were considered due to the likelihood of subgroups having different dietary preferences and intake levels compared to the overall sample. The choice of 65 years as the age threshold and 30 kg/m^2^ as the BMI threshold aligns with the World Health Organization (WHO) guidelines for defining elderly and obese populations in developed countries, recognizing the unique health challenges associated with increasing age and BMI.

To minimize the potential impact of reverse causation, we performed sensitivity analyses by excluding participants who experienced cardiovascular events within the first 2 years of the follow-up period. Additionally, we only included data from participants who had completed three or more online 24-hour dietary recall assessments to ensure a more accurate representation of their typical dietary habits.

All statistical analyses were performed using Python (version 3.9.13) with scikit-learn^[21]^ and R (version 4.1.2). A two-sided *P* value of less than .05 was deemed statistically significant.

## 3. Results

### 3.1. Baseline characteristics

During a median follow-up of 11.7 years (917,477 person-years), 9806 participants developed CVD, including 4518 with CAD and 1258 with stroke. The characteristics of all participants and those who developed CVD are presented in Table 1. On average, those who developed CVD were 4.3 years older than the former group. In terms of sex and ethnicity, 40,214 participants (44.60%) were male and 86,961 (96.44%) were white. Individuals with CVD were more likely to be male, less educated, have a higher BMI and SBP, compared to females who were more educated, had a lower BMI and SBP. Moreover, they were more likely to be current smokers than nonsmokers and, if they drank alcohol, to consume over 20 g/d. The characteristics of those who developed CAD and stroke are respectively presented in Table S1, Supplemental Digital Content, http://links.lww.com/MD/N342 and Table S2, Supplemental Digital Content, http://links.lww.com/MD/N342.

**Table 1:** Characteristics of the study cohort, by incident case status for CVD in UK Biobank.*

Characteristics	Total	Non-CVD	CVD
Age, yr, mean ± SD	58.50 ± 7.85	58.07 ± 7.82	62.39 ± 6.95
Sex, n (%)			
Female	49,953 (55.40)	56,247 (56.88)	3706 (41.84)
Male	40,214 (44.60)	35,063 (43.12)	5151 (58.16)
Ethnicity, n (%)			
White	86,961 (96.44)	78,364 (96.38)	8597 (97.06)
Non-White	3206 (3.56)	2946 (3.62)	260 (2.94)
Education years, n%			
≤7 years	4683 (5.19)	3958 (4.87)	725 (8.19)
10–13 years	17,707 (19.64)	15,925 (19.59)	1782 (20.12)
15–20 years	67,777 (75.17)	61,427 (75.55)	6350 (71.69)
BMI categories, n (%)			
Underweight and Normal weight (<25)	36,689 (40.69)	33,801 (41.57)	2888 (32.61)
Overweight (25 to < 30)	36,708 (40.71)	32,905 (40.47)	3803 (42.94)
Obese ≥ 30	16,770 (18.60)	14,604 (17.96)	2166 (24.46)
Smoking, n (%)			
Never	52,594 (58.33)	48,142 (59.21)	4452 (50.27)
Previous	31,183 (34.58)	27,527 (33.85)	3656 (41.28)
Current	6390 (7.09)	5641 (6.94)	749 (8.46)
Alcohol intake, n (%)			
Nondrinker	21,360 (23.69)	19,222 (23.64)	2138 (24.14)
0–< 10 g/d	22,141 (24.56)	20,085 (24.70)	2056 (23.21)
10–< 20 g/d	17,798 (19.74)	16,209 (19.93)	1589 (17.94)
≥20 g/d	28,868 (32.02)	25,794 (31.72)	3074 (34.71)
Physical activity, n (%)			
Low	22,298 (24.73)	20,128 (24.75)	2170 (24.50)
Moderate	47,361 (52.53)	42,860 (52.71)	4501 (50.82)
High	20,508 (22.74)	18,322 (22.53)	2186 (24.68)
SBP, n (%)			
<90 mm Hg	23 (0.03)	21 (0.03)	2 (0.02)
90–<140 mm Hg	56,470 (62.63)	52,066 (64.0)	4404 (49.72)
≥140 mm Hg	33,674 (37.35)	29,223 (35.94)	4451 (50.25)
Vitamin C, mg	151.64 ± 90.32	151.6 ± 89.97	152.3 ± 93.47
Vitamin B6, mg	2.16 ± 0.62	2.16 ± 0.62	2.23 ± 0.65
Vitamin B12, mg	6.50 ± 3.71	6.48 ± 3.69	6.69 ± 3.82
Vitamin E, mg	9.43 ± 4.00	9.44 ± 3.98	9.35 ± 4.20
Vitamin D, mg	2.87 ± 2.24	2.86 ± 2.23	2.97 ± 2.29
Folate, ug	302.66 ± 97.31	301.8 ± 96.90	310.6 ± 100.7
Retinol, ug	329.87 ± 151.87	329.2 ± 151.5	336.3 ± 155.1
Potassium, mg	3778.45 ± 1094.70	3769.3 ± 1084.3	3862.1 ± 1183.0
Magnesium, mg	350.97 ± 95.21	350.4 ± 94.77	356.4 ± 98.96
Iron, mg	13.76 ± 3.81	13.74 ± 3.79	14.03 ± 3.96
Calcium, mg	981.19 ± 355.20	979.4 ± 333.0	997.3 ± 354.8

* Values are mean (standard deviation) or number (percentages) unless otherwise indicated.

CVD = cardiovascular disease, SD = standard deviation.

### 3.2. Epidemiological association of micronutrient intake with CVD

Table 2 illustrates the relationship between CVD and the intake levels of 11 micronutrients, using the lowest-intake group as a reference point. Vitamin E intake displayed the strongest inverse correlation with CVD risk across models 1, 2, and 3. The odds ratios (ORs) with 95% confidence intervals (95% CIs) for vitamin E intake in groups 2, 3, and 4 compared to group 1 were 0.82 (0.77–0.88), 0.79 (0.74–0.85), and 0.77 (0.72–0.82) respectively in model 3. Furthermore, higher potassium intake in the multivariable models was associated with a reduced risk of CVD events, with multivariable ORs and 95% CIs for groups 2, 3, and 4 at 0.89 (0.83–0.95), 0.87 (0.81–0.93), and 0.88 (0.80–0.96) respectively in model 3. Moreover, vitamin C and calcium intake in the multivariable models exhibited a significant inverse relationship with CVD risk, with corresponding ORs and 95% CIs at 0.90 (0.84–0.96) and 0.91 (0.85–0.97) for the highest-intake levels in model 3. The linear association between daily intake of micronutrients and risk of CVD is illustrated in Figure S10, Supplemental Digital Content, http://links.lww.com/MD/N342, which indicates that, except for vitamin B6 and vitamin B12, there are non-linear associations between micronutrients and CVD.

**Table 2:** Multivariable-adjusted odds ratios and 95% confidence intervals for dietary micronutrient intakes with the risk of CVD events in the study cohort.

	Quartile of Nutrient Intake				*P* for trend
	Q1	Q2 OR (95% CI)*	Q3 OR (95% CI)	Q4 OR (95% CI)	
Vitamin B6					
Expose	1.63 (0.12, 1.83)	1.99 (1.83, 2.14)	2.3 (2.14, 2.48)	2.74 (2.48, 6.39)	
No. of cases	2358/25514	2525/25513	2494/25514	2807/25513	
Model 1†	Ref.	1.08 (1.01–1.16)	1.06 (0.99–1.13)	1.22 (1.14–1.3)	<.01
Model 2‡	Ref.	0.99 (0.93–1.06)	0.93 (0.87–1)	1.02 (0.95–1.09)	.979
Model 3§	Ref.	1 (0.94–1.07)	0.94 (0.88–1.01)	1.02 (0.96–1.1)	.872
Vitamin B12					
Expose	3.16 (0.01, 4.08)	4.89 (4.08, 5.75)	6.81 (5.75, 8.26)	10.49 (8.26, 46.46)	
No. of cases	2486/25514	2522/25513	2521/25514	2655/25513	
Model 1	Ref.	1.03 (0.97–1.1)	1.02 (0.96–1.09)	1.09 (1.02–1.16)	.021
Model 2	Ref.	0.96 (0.9–1.03)	0.92 (0.86–0.99)	0.94 (0.88–1)	.034
Model 3	Ref.	0.97 (0.9–1.03)	0.94 (0.88–1)	0.96 (0.89–1.02)	.140
Folate					
Expose	216.72 (38.53, 245.7)	269.19 (245.71, 291.71)	315.78 (291.71, 345.86)	391.34 (345.87, 1195.69)	
No. of cases	2447/25514	2416/25513	2610/25514	2711/25513	
Model 1	Ref.	0.98 (0.92–1.05)	1.07 (1–1.15)	1.13 (1.06–1.21)	<.01
Model 2	Ref.	0.92 (0.86–0.98)	0.97 (0.91–1.04)	1 (0.94–1.07)	.572
Model 3	Ref.	0.93 (0.87–1)	0.99 (0.92–1.06)	1.01 (0.94–1.08)	.377
Iron					
Expose	9.59 (0.96, 10.73)	11.58 (10.73, 12.38)	13.22 (12.38, 14.18)	15.58 (14.18, 33.07)	
No. of cases	2504/25514	2478/25513	2529/25514	2673/25513	
Model 1	Ref.	0.99 (0.93–1.06)	1.02 (0.96–1.09)	1.09 (1.02–1.16)	<.01
Model 2	Ref.	0.9 (0.84–0.97)	0.91 (0.85–0.98)	0.96 (0.9–1.02)	.343
Model 3	Ref.	0.92 (0.86–0.99)	0.94 (0.88–1)	0.98 (0.91–1.05)	.766
Vitamin C					
Expose	63.37 (0.11, 88.29)	110.74 (88.29, 133.9)	160.19 (133.9, 192.75)	244.58 (192.75, 1280.72)	
No. of cases	2618/25514	2538/25513	2567/25514	2461/25513	
Model 1	Ref.	0.95 (0.89–1.01)	0.98 (0.92–1.04)	0.94 (0.88–1)	.109
Model 2	Ref.	0.89 (0.83–0.95)	0.9 (0.84–0.96)	0.87 (0.81–0.93)	<.01
Model 3	Ref.	0.91 (0.85–0.98)	0.93 (0.87–1)	0.91 (0.85–0.97)	.014
Potassium					
Expose	2996.2 (497.82, 3301.29)	3527.57 (3301.31, 3735)	3945.94 (3735.01, 4192.29)	4552.4 (4192.32, 9157.98)	
No. of cases	2513/25514	2488/25513	2535/25514	2648/25513	
Model 1	Ref.	0.97 (0.9–1.03)	1.01 (0.94–1.07)	1.07 (1–1.14)	.026
Model 2	Ref.	0.87 (0.82–0.93)	0.85 (0.8–0.91)	0.86 (0.81–0.92)	<.01
Model 3	Ref.	0.89 (0.83–0.95)	0.87 (0.81–0.93)	0.88 (0.83–0.95)	<.01
Magnesium					
Expose	284.96 (94.37, 309.95)	328.78 (309.95, 345.67)	363.17 (345.67, 384.08)	413.71 (384.08, 746.97)	
No. of cases	2616/25514	2475/25513	2526/25514	2567/25513	
Model 1	Ref.	0.95 (0.89–1.01)	0.99 (0.93–1.06)	1.01 (0.95–1.08)	.400
Model 2	Ref.	0.88 (0.82–0.94)	0.9 (0.84–0.96)	0.92 (0.86–0.98)	.036
Model 3	Ref.	0.9 (0.84–0.96)	0.92 (0.86–0.98)	0.93 (0.87–1)	.117
Retinol					
Expose	189.7 (0.18, 238.76)	278.41 (238.76, 316.55)	357.41 (316.56, 406.51)	476.88 (406.51, 1016.49)	
No. of cases	2558/25514	2539/25513	2465/25514	2622/25513	
Model 1	Ref.	1.02 (0.95–1.09)	0.98 (0.92–1.05)	1.04 (0.97–1.11)	.447
Model 2	Ref.	0.99 (0.93–1.06)	0.93 (0.87–1)	0.96 (0.9–1.03)	.084
Model 3	Ref.	1.01 (0.94–1.08)	0.95 (0.88–1.01)	0.98 (0.91–1.04)	.201
Vitamin D					
Expose	1.02 (0, 1.42)	1.79 (1.42, 2.23)	2.83 (2.23, 3.79)	5.37 (3.79, 26.44)	
No. of cases	2395/25515	2596/25512	2593/25514	2600/25513	
Model 1	Ref.	1.03 (0.97–1.1)	1.07 (1–1.14)	1.07 (1–1.14)	.031
Model 2	Ref.	0.97 (0.91–1.04)	0.97 (0.9–1.03)	0.94 (0.88–1)	.074
Model 3	Ref.	0.97 (0.91–1.04)	0.97 (0.91–1.04)	0.95 (0.89–1.02)	.186
Calcium					
Expose	721.64 (0.75, 818.65)	892.21 (818.67, 960.28)	1030.7 (960.28, 1114.52)	1236.97 (1114.53, 2759.87)	
No. of cases	2675/25514	2420/25513	2541/25514	2548/25513	
Model 1	Ref.	0.87 (0.81–0.93)	0.94 (0.88–1)	0.94 (0.89–1.01)	.323
Model 2	Ref.	0.84 (0.79–0.9)	0.89 (0.83–0.95)	0.88 (0.82–0.94)	.002
Model 3	Ref.	0.86 (0.8–0.92)	0.91 (0.85–0.97)	0.9 (0.84–0.96)	.015
Vitamin E					
Expose	5.22 (0.01, 6.36)	7.25 (6.36, 8.06)	8.92 (8.06, 9.96)	11.5 (9.96, 31.2)	
No. of cases	2962/25514	2530/25513	2444/25514	2248/25513	
Model 1	Ref.	0.82 (0.77–0.87)	0.78 (0.73–0.83)	0.73 (0.69–0.78)	<.01
Model 2	Ref.	0.8 (0.75–0.86)	0.77 (0.72–0.82)	0.74 (0.69–0.79)	<.01
Model 3	Ref.	0.82 (0.77–0.88)	0.79 (0.74–0.85)	0.77 (0.72–0.82)	<.01

* Linear logistic regression models were used to estimate ORs and 95% CIs.

† Model 1 did not adjust for any confounders.

‡ Model 2 adjusted for age (continuous), sex (male, female).

§ Model 3 additionally adjusted for age (continuous), sex (male, female), ethnicity (white subjects, non-white subjects), BMI (kg/m2; <25.0, 25.0–29.9, ≥30.0), income (pounds/year; <18,000, 18,000–30,999, 31,000-51,999, ≥52,000), smoking status (never, previous, current), alcohol intake (g/d; nondrinker, 0–10, 10–20, ≥20), physical activity (min/day; low: <15, moderate: 15–75, high: ≥75), education year (year; 0–7, 10–13, 15–20) and personal medical history of hypertension and diabetes (yes or no).

### 3.3. Pearson correlations

**Figure 1.**
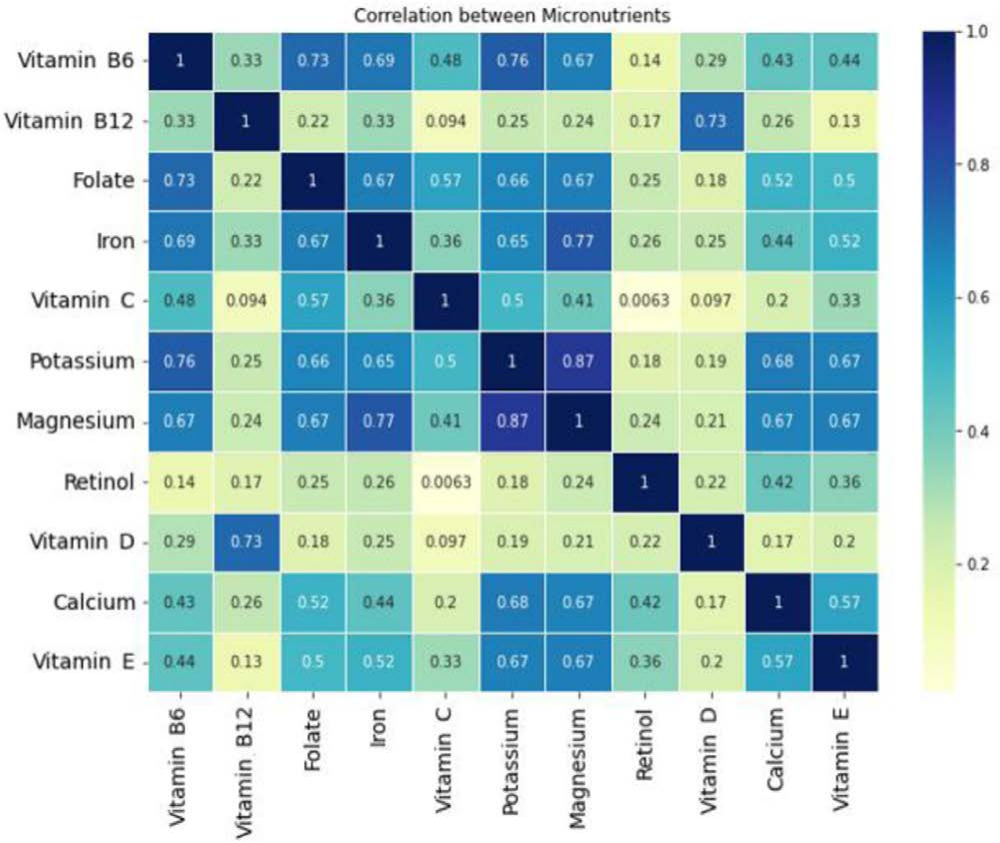
Heat map of Pearson correlation coefficients. The darker the color is on the hot map, the stronger the linear correlation between two features.

Figure 1 showcases a heat map depicting the Pearson correlation coefficients between different micronutrients. Strong linear relationships, with Pearson correlation coefficients greater than or equal to 0.7, are observed between vitamin B6 and folate, vitamin B6 and potassium, vitamin B12 and vitamin D, magnesium and iron, as well as potassium and magnesium.

### 3.4. Model selection

**Figure 2.**
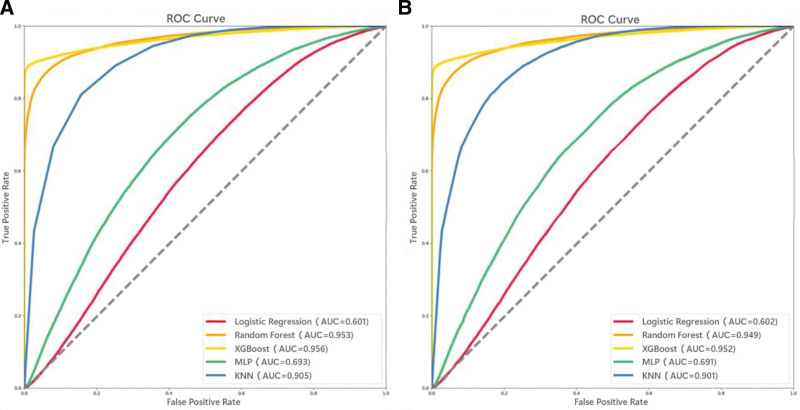
The area under the ROC curves for different machine learning algorithms were generated for both the training (A) and testing (B) sets, and the legend shows the ROC curve. ROC = receiver operator characteristic.

Performance and reliability of machine learning models were evaluated using accuracy, recall, specificity, and F1-score, as detailed in Table 3. Following a grid search, the XGBoost model’s optimal hyperparameters were determined as 1000 trees with a learning rate of 0.1 and a tree depth of 3. This configuration exhibited the best performance based on evaluation criteria and displayed superior discriminatory abilities on both training and testing sets. Figure 2 illustrates the receiver operator characteristic curves for the models, indicating that the XGBoost model achieved the highest area under the receiver operating characteristic curve (AUC) (0.956) on the training set, followed by the RF model (0.953). On the testing set, the XGBoost model surpassed all other models with an AUC of 0.952. Feature importance analysis was conducted using the XGBoost model due to its superior predictive performance and operational characteristics.

**Table 3:** The comparison of the performance of various machine learning models.

Model	Training Datasets				Testing Datasets			
	Accuracy	Precision	Recall	F1-score	Accuracy	Precision	Recall	F1-score
LR	0.581	0.58	0.58	0.58	0.579	0.58	0.58	0.58
XGBoost	**0.926**	**0.94**	**0.93**	**0.93**	**0.925**	**0.93**	**0.93**	**0.93**
KNN	0.809	0.85	0.81	0.80	0.773	0.83	0.77	0.76
MLP	0.521	0.67	0.62	0.59	0.519	0.66	0.62	0.59
RF	**0.921**	**0.93**	**0.91**	**0.91**	**0.913**	**0.92**	**0.91**	**0.91**

The bold values indicate the optimum model parameter.

KNN = k-nearest neighbors, MLP = multilayer perceptron algorithm, LR = logistic regression, XGBoost = eXtreme gradient boosting.

**Figure 3.**
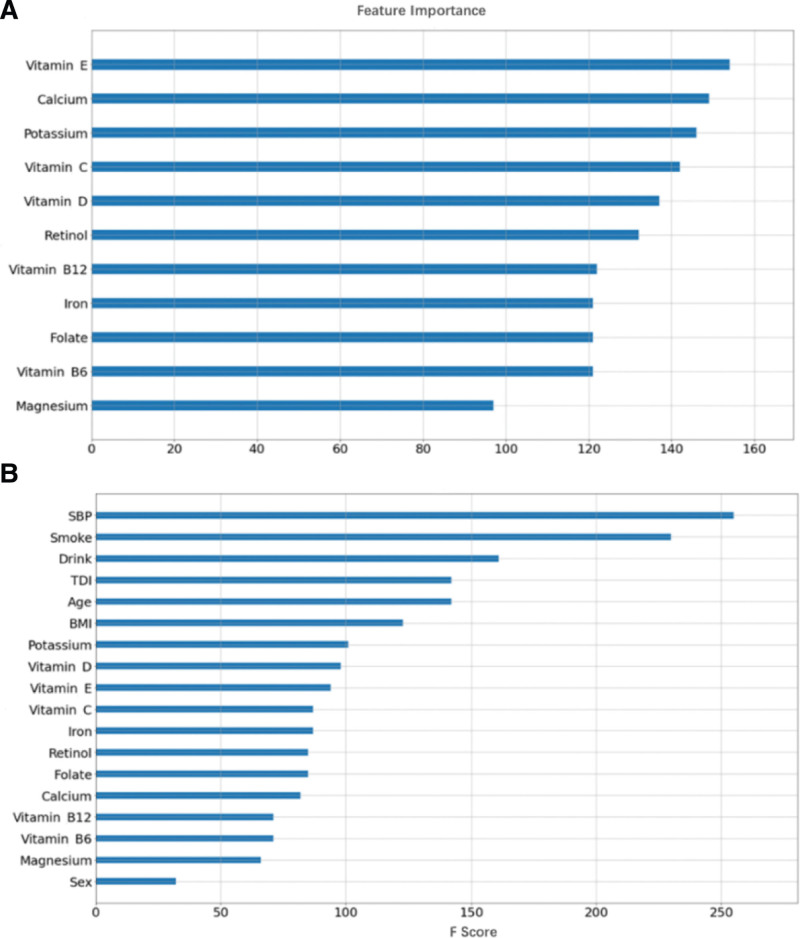
Relative micronutrient feature importance for risk to CVD. (A) Without covariates. (B) Covariates included age(continuous), sex (male, female), ethnicity (white subjects, nonwhite subjects), BMI (continuous), smoking status (never, previous, current), alcohol intake(continuous), TDI (continuous), SBP (continuous). The calculation method of feature importance in the XGBoost model is the weight (the number of times a feature is used to split the data across all trees). BMI = body mass index, CVD = cardiovascular disease, TDI = Townsend deprivation index, XGBoost = eXtreme gradient boosting.

Figure 3 depicts the key features highlighted by the XGBoost model. In Figure 3A, the top five crucial micronutrient features were identified as vitamin E, calcium, potassium, vitamin C, and vitamin D intake. Additionally, Figure 3B showcases the significance of features in the model, which encompassed 11 micronutrients and seven common predictors. Notably, long-term risk factors such as SBP, smoking, drinking, TDI, age, and BMI were deemed more important predictors of CVD risk compared to micronutrient intake.

### 3.5. Secondary outcomes

The study examined 4090 cases of CAD and 1132 cases of stroke as secondary cardiovascular outcomes. Analysis of micronutrient intake indicated that the importance of each micronutrient for predicting CAD events was similar to that for CVD events. Potassium, vitamin D, vitamin C, and vitamin E intake were significant predictors of CAD events (Figure S2A, Supplemental Digital Content, http://links.lww.com/MD/N342). In contrast, the top 5 factors predicting stroke events were potassium, magnesium, iron, vitamin C, and vitamin B12 intake (Figure S2B, Supplemental Digital Content, http://links.lww.com/MD/N342).

### 3.6. Stratification and sensitivity analyses

Subgroup analyses were conducted to investigate the association between the intake of each micronutrient and CVD risk, stratified by age, sex, BMI, and smoking status. These analyses confirmed a strong overall association between CVD risk and potassium, vitamin E, and vitamin C intake (Figure S3–6, Supplemental Digital Content, http://links.lww.com/MD/N342). Moreover, calcium intake was found to be significantly associated with CVD risk in older adults (≥65 years) and obese adults. Our results also revealed a gender-specific difference in the association between vitamin B6 intake and CVD risk, showing a strong association in women. However, the effect of smoking status on the association between micronutrients and the risk of CVD was minimal.

Sensitivity analyses were performed on participants who completed three or more surveys (N = 61,564) or did not have cancer (N = 87,181). Results indicated that these groups had similar importance in relation to the top nutrient intake features as observed in the main analysis (Figures S7 and S8, Supplemental Digital Content, http://links.lww.com/MD/N342). Comparable findings were also seen when excluding the first 2 years of follow-up (n = 89,020) (Figure S9, Supplemental Digital Content, http://links.lww.com/MD/N342).

## 4. Discussion

This study utilized machine learning algorithms to create CVD prediction models based on the consumption of 11 micronutrients and 7 common CVD predictors. The XGBoost model demonstrated superior performance on both the training and testing datasets, achieving AUCs of 0.956 and 0.952, respectively. Consequently, the XGBoost model was employed to assess the importance of features, revealing that across all intake level groups, potassium, vitamin E, and vitamin C were crucial micronutrients for predicting CVD risk. Furthermore, the study confirmed that long-term risk factors such as high SBP, smoking, alcohol consumption, and obesity were more significant predictors of CVD risk compared to nutrient intake.

Machine learning has been utilized in studies to create CVD risk prediction models that consider both food and nutrient factors. For example, the ATTICA study demonstrated that machine learning methods based on food and nutrient factors outperformed traditional statistical techniques in classifying CVD samples.^[22]^ Similarly, a Canadian study developed machine learning models incorporating 61 nutrition variables and 14 socioeconomic variables, revealing varied associations between nutrient intake and CVD risk. The study highlighted that the consumption of vitamin or mineral supplements, caffeine intake from food or drink, and alcohol had the most significant impact on predicting CVD risk.^[23]^

Three logistic regression models were developed to investigate the relationship between micronutrient intake and the risk of CVD. Model 1 was used as an unadjusted baseline model to capture the initial association between CVD risk and micronutrient intake. Model 2 included adjustments for age and sex, as these variables were identified as important confounding factors that could influence the observed associations. The rationale for these adjustments was to account for demographic influences that could potentially affect the accuracy of our results. Model 3 represents the fully adjusted model, where a comprehensive set of confounders was considered, including age, sex, ethnicity, BMI, income, smoking status, alcohol intake, physical activity, years of education, and personal medical history of hypertension and diabetes. This exhaustive adjustment aimed to refine our understanding of the relationships by accounting for potential interactions and interdependencies among multiple factors. In Model 1, the unadjusted model, vitamin B6, B12, folate, and iron showed significant associations with the outcome, suggesting a potential role in CVD risk. However, in Model 2 and the fully adjusted Model 3, these associations lost statistical significance. The loss of significance in Model 2 and Model 3 indicates that the initial associations observed in Model 1 may have been influenced or confounded by other factors that were subsequently controlled for. On the other hand, for vitamin C and calcium, the change in significance from nonsignificant in Model 1 to significant in Model 2 and Model 3 highlights the importance of considering multiple factors simultaneously. These findings underscore the complexity of nutritional epidemiology and the multifaceted nature of relationships between micronutrient intake and health outcomes. It is important to acknowledge that nutritional factors are inherently interconnected with various lifestyle and demographic variables. The adjustments made in the fully adjusted Model 3 aimed to disentangle these complex relationships, however, the nuanced interplay between nutrients and confounders presents challenges in isolating their individual effects. We believe that machine learning techniques have the potential to enhance the analysis of complex relationships between nutrients and health outcomes.

The XGBoost model focused on micronutrients identified vitamin E, calcium, potassium, and vitamin C intake as the top predictors of CVD risk in a large UK population cohort. These results align with the ORs from multiple logistic regression analysis. Potassium intake consistently stood out as the most significant predictor of CVD risk across all analyses, emphasizing its vital role in cardiovascular health. Various studies have supported the blood pressure-lowering effects of potassium intake, suggesting its potential in reducing CVD risk related to hypertension.^[24]^ Our study revealed that participants with a potassium intake of around 2500–4300 mg/d had the lowest risk of CVD, aligning closely with the European Food Safety Authority (EFSA) recommended intake of 3500 mg/d.

Vitamin E and vitamin C are antioxidants, and their intake has consistently shown an association with CVD risk across all groups. This relationship may be attributed to the role of these nutrients in reducing cholesterol-induced atherosclerotic lesions,^[25,26]^ decreasing lesion size, and improving serum lipid profiles.^[27]^ In our cohort, the recommended daily intakes for vitamin E and vitamin C are around 10 and 250 mg, respectively.

Calcium intake has been identified as a significant predictor of CVD risk in adults with a BMI of 30.0 or higher. Numerous epidemiological studies have demonstrated a correlation between obesity or high BMI and an increased risk of CVD events, while also revealing a notable inverse relationship between calcium intake and body fat percentage.^[28–30]^ This suggests that BMI may play a mediating role in the link between calcium intake and CVD.^[31]^ Furthermore, our research indicates that the relationship between calcium intake and CVD risk is more pronounced in older adults compared to younger adults, suggesting that a moderate increase in calcium-rich foods consumption could potentially help prevent CVD in individuals aged 65 and above. This finding is consistent with a European study on older adults, which proposed that boosting daily calcium intake could positively influence the aging process and reduce the risk of chronic diseases.^[32]^

The association between the intake of B vitamins (vitamin B6, vitamin B12, and folate) and CVD risk, as investigated in previous studies,^[33–35]^ was found to be significant only among women in the present study. This indicates that incorporating vitamin B6 supplementation into women’s daily diets could potentially reduce their risk of CVD. It is important to note that our XGBoost model did not rely on assumptions regarding interactions or variable linearity, therefore, the significance of these micronutrients in relation to CVD risk should be considered within a broader context.

This study stands out for its utilization of a machine learning algorithm to investigate intricate relationships without assuming linearity between micronutrient intake and CVD risk. While previous research has made valuable contributions to identifying risk factors for CVD or other metabolic diseases using machine learning algorithms,^[36–39]^ our study uniquely focuses on uncovering the connection between micronutrient intake and CVD risk. In the dynamic field of CVD research, studies have predominantly concentrated on pinpointing risk factors and developing high-performance prediction models through advanced computational techniques. Our approach sets itself apart by delving into the specific realm of micronutrient intake, providing insights into how these dietary elements may impact CVD risk. By employing a dual methodology that combines traditional logistic regression models with explainable machine learning, we not only navigate the intricacies of non-linear relationships but also offer interpretable insights into the role of micronutrients in cardiovascular health. Furthermore, our analysis drew on comprehensive data regarding sociodemographic characteristics, lifestyle factors, and potential confounding variables from a large-scale, long-term cohort. This enabled us to control for confounding variables and investigate the relationship between micronutrient intake and CVD risk.

Limitations of this study include its observational design, which precludes establishing causality and leaves room for residual confounding. Caution is advised when interpreting the findings. Additionally, reliance on self-reported dietary assessments introduces the risk of recall bias and misreporting, potentially impacting the accuracy of estimates. To mitigate this, we utilized estimated nutrient data from a minimum of two 24-hour dietary assessments per participant. The study also did not account for the potential influence of multivitamin or mineral supplement use on the results. Furthermore, the analysis was constrained by the absence of data on the intake of other essential micronutrients like zinc, sodium, and selenium, limiting the breadth of the analysis.

## 5. Conclusion

In this comprehensive cohort study involving UK Biobank participants, machine learning models were utilized to forecast the likelihood of CVD based on micronutrient intake data. The results revealed that the XGBoost model outperformed other models (KNN, MLP, logistic regression) in terms of predictive accuracy. Notably, potassium, vitamin E, and vitamin C intake emerged as significant predictors of CVD in the adult population. Our findings recommend that older and obese individuals consider increasing their consumption of calcium-rich foods, while women may benefit from boosting their intake of vitamin B6-rich foods. Furthermore, our study suggests that targeted interventions focusing on addressing long-term risk factors such as SBP, smoking, alcohol consumption, and obesity may be more effective in reducing CVD-related morbidity and mortality compared to daily micronutrient supplementation for CVD prevention.

## Author contributions

**Conceptualization:** Hongpeng Sun, Liyuan Han.

**Data curation:** Hongpeng Sun, Liyuan Han.

**Formal analysis:** Yue Wang.

**Funding acquisition:** Shiliang Ling, Yuyi Sha.

**Investigation:** Yue Wang.

**Methodology:** Yue Wang.

**Project administration:** Shiliang Ling, Yuyi Sha.

**Validation:** Hongpeng Sun, Shiliang Ling.

**Writing – original draft:** Yue Wang.

**Writing – review & editing:** Hongpeng Sun, Liyuan Han.

## Supplementary Material
