# Supplementary material for: Dietary intake of potassium, vitamin E, and vitamin C emerges as the most significant predictors of cardiovascular disease risk in adults
Source: Medicine (Baltimore). 2024 Aug 9;103(32):e39180. doi: 10.1097/MD.0000000000039180 (PMC11315499; doi:10.1097/MD.0000000000039180)
Supplement: Supplementary file 1 [file medi-103-e39180-s001.pdf]

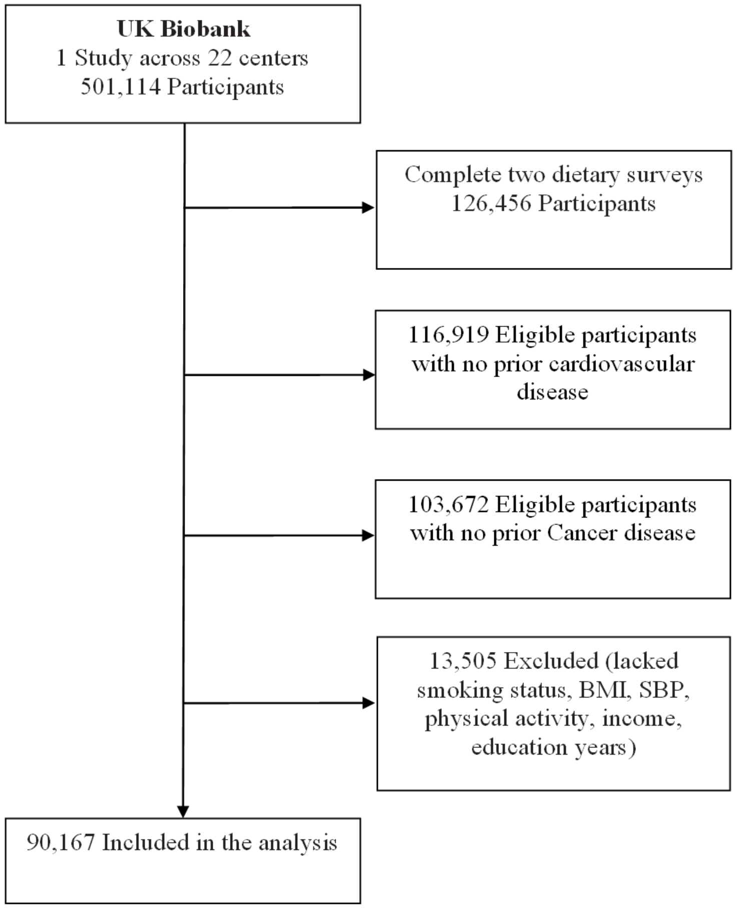

Supplementary Figure 1 Flowchart of the participants selection.

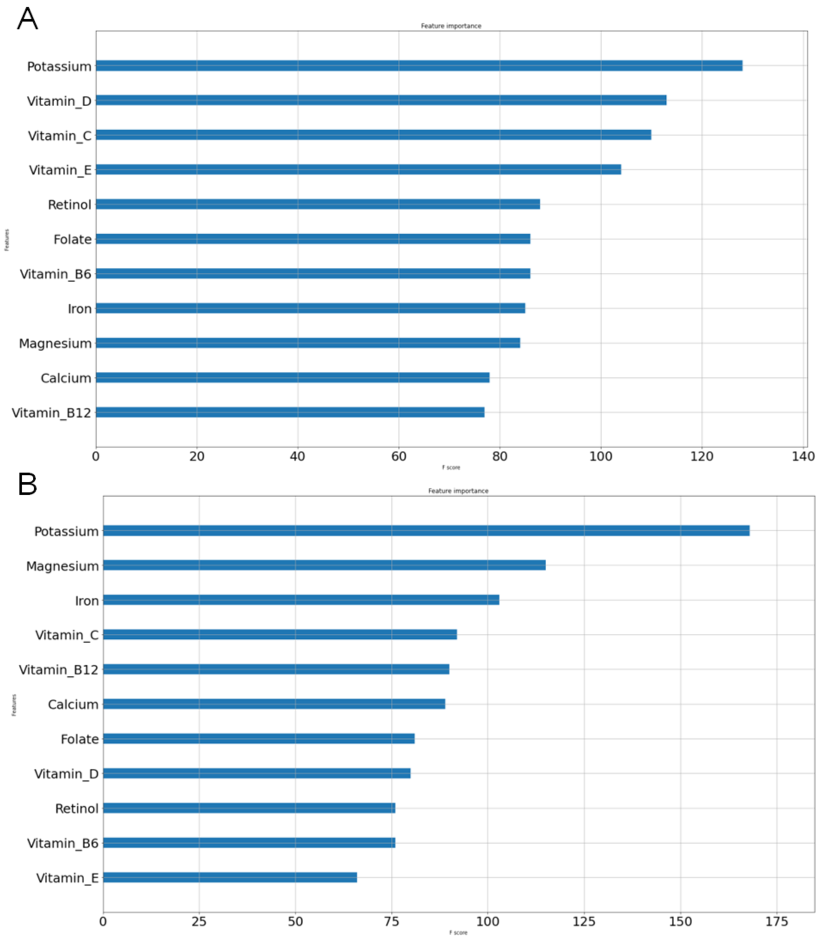

Supplementary Figure 2 Relative feature importance for risk to CAD (A) and stroke (B). We estimated feature importance using the permutation method and used a logarithmic scale for better visualization.

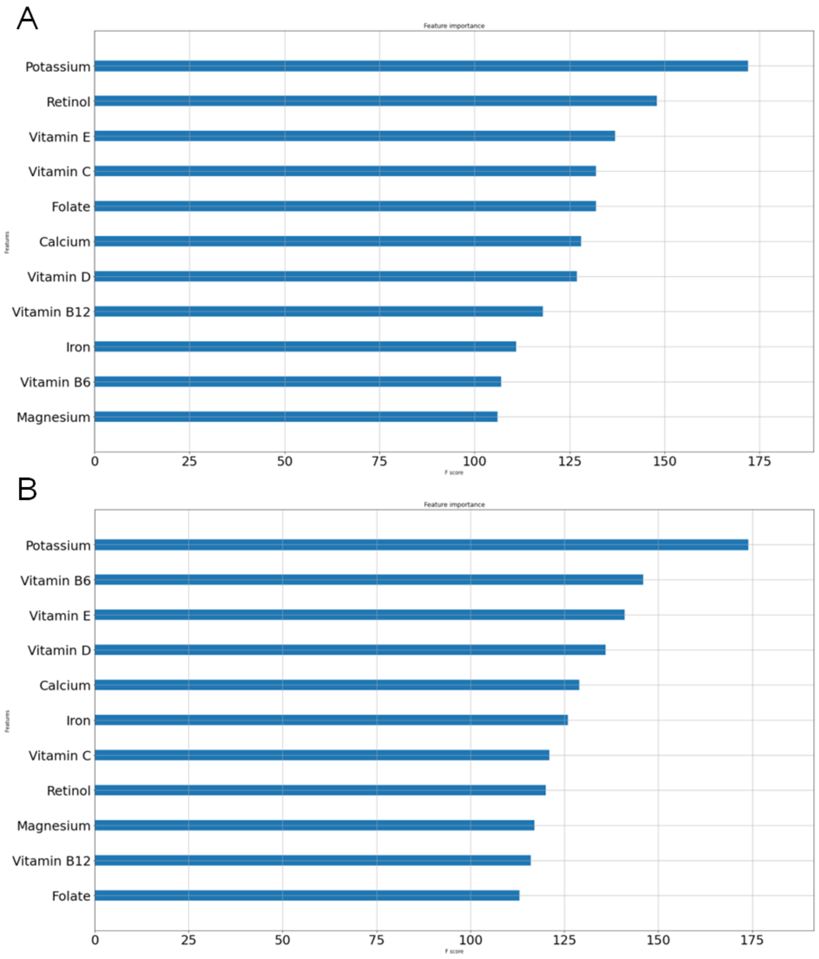

Supplementary Figure 3 Relative feature importance for risk to CVD, stratified by sex. (A)male, (B)female. We estimated feature importance using the permutation method and used a logarithmic scale for better visualization.

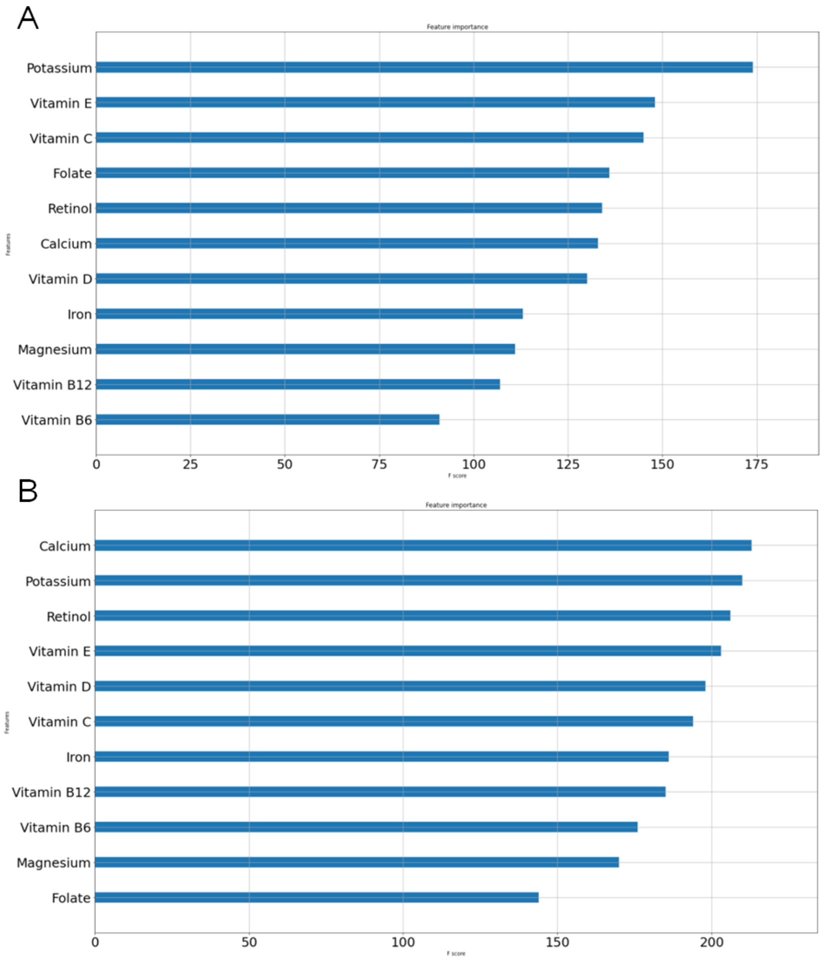

Supplementary Figure 4 Relative feature importance for risk to CVD, stratified by age (year). (A) <65, (B) ≥65. We estimated feature importance using the permutation method and used a logarithmic scale for better visualization.energy. Shaded areas represent 95% confidence

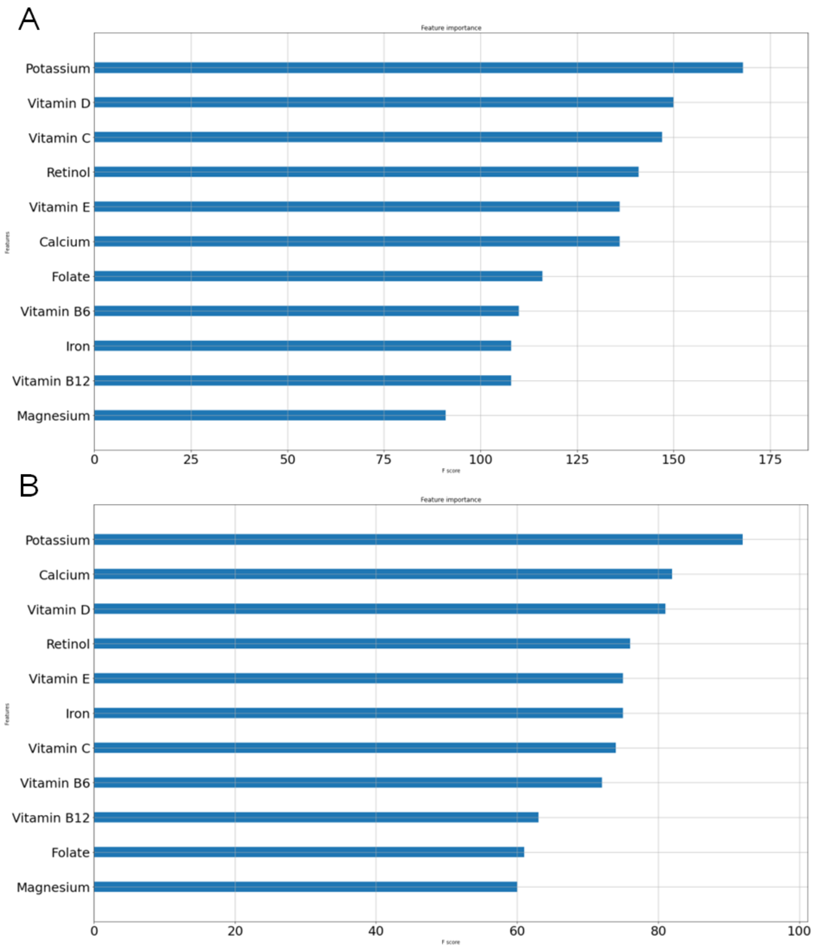

Supplementary Figure 5 Relative feature importance for risk to CVD, stratified by BMI (kg/m^2). (A) <30.0, (B) ≥30.0. We estimated feature importance using the permutation method and used a logarithmic scale for better visualization.

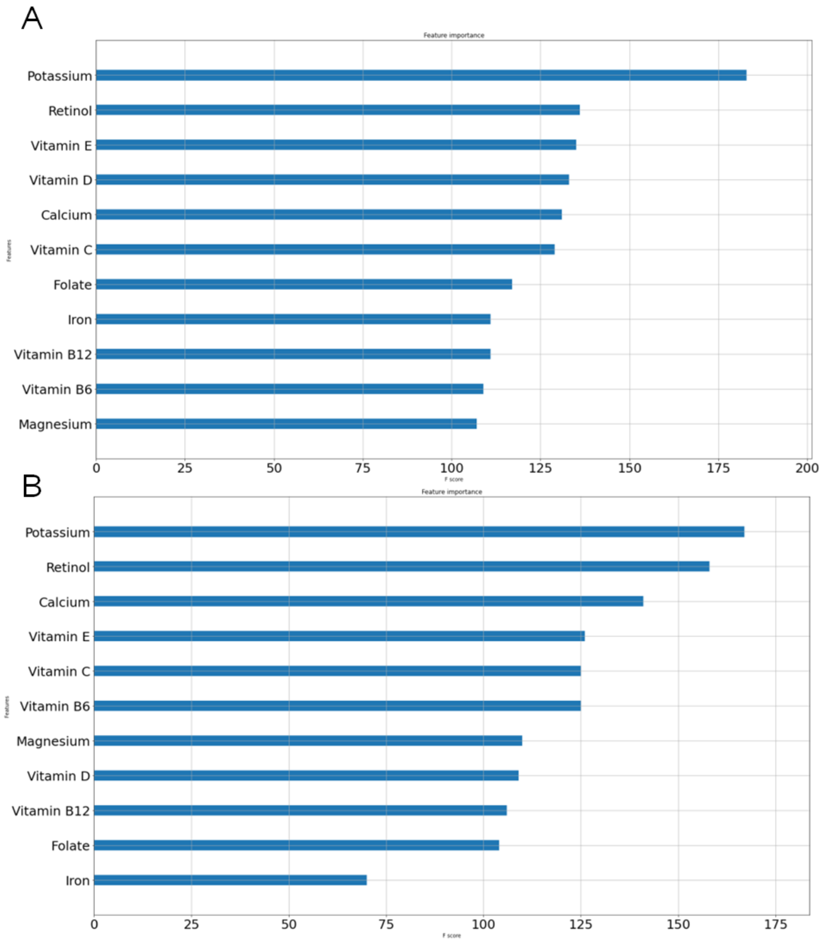

Supplementary Figure 6 Relative feature importance for risk to CVD, stratified by smoking. (A) never, (B) current. We estimated feature importance using the permutation method and used a logarithmic scale for better visualization.

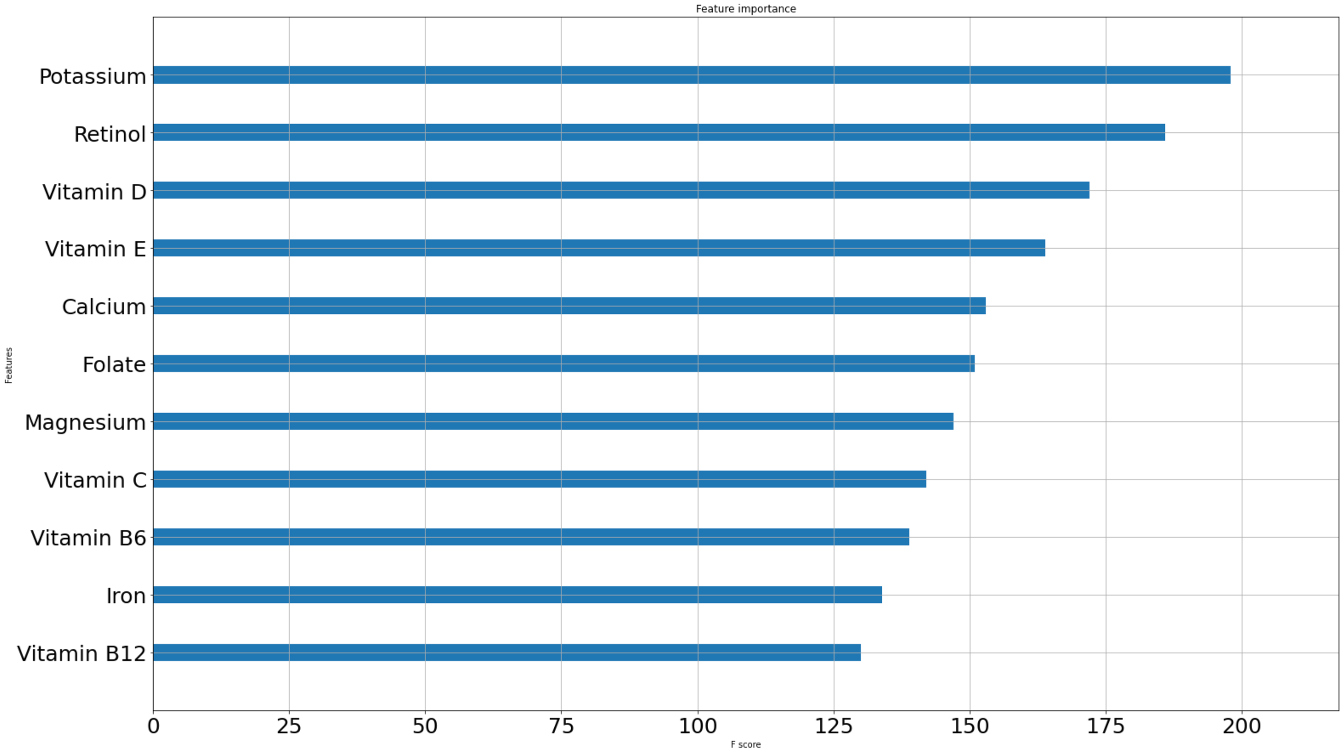

Supplementary Figure 7 Relative feature importance for risk to CVD, excluding participates who completed the survey on two occasions. We estimated feature importance using the permutation method and used a logarithmic scale for better visualization.

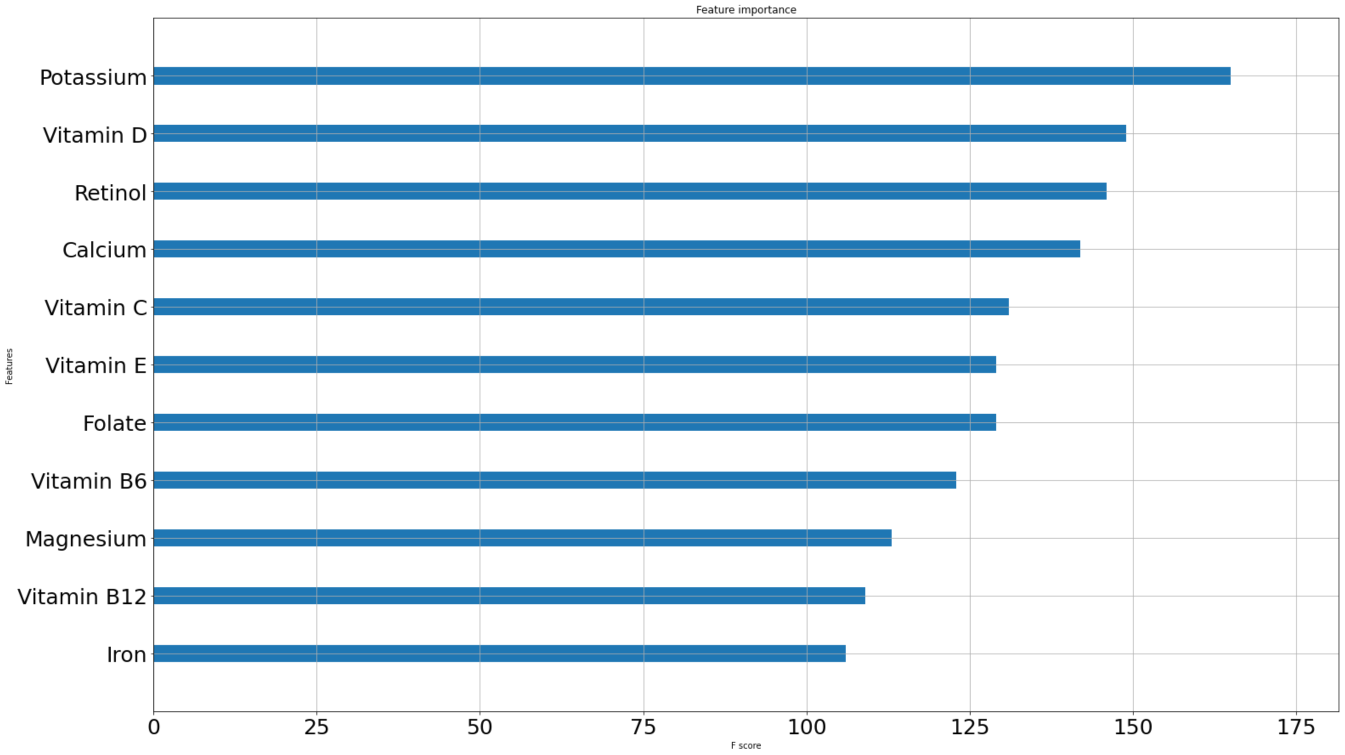

Supplementary Figure 8 Relative feature importance for risk to CVD, excluding participates with cancer. We estimated feature importance using the permutation method and used a logarithmic scale for better visualization.

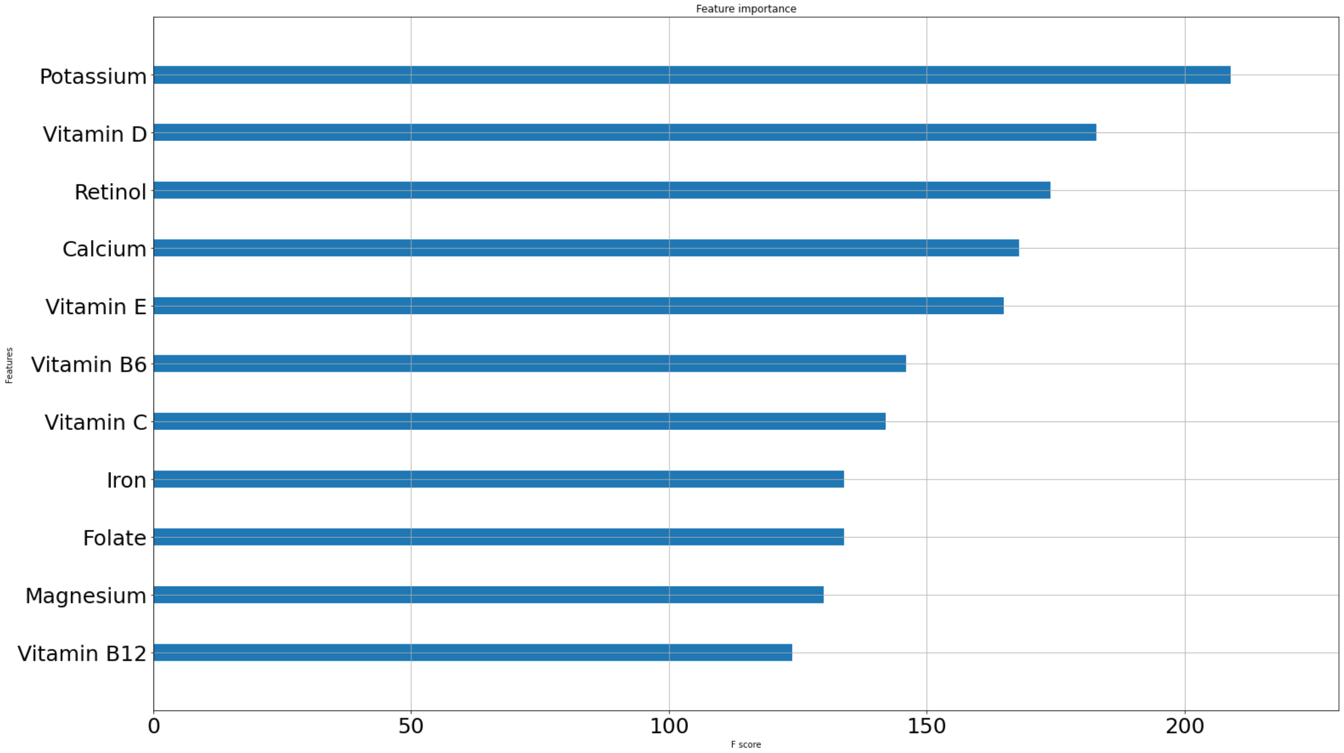

Supplementary Figure 9 Relative feature importance for risk to CVD, excluding participates who developed CVD events during the first two years of follow-up. We estimated feature importance using the permutation method and used a logarithmic scale for better visualization.

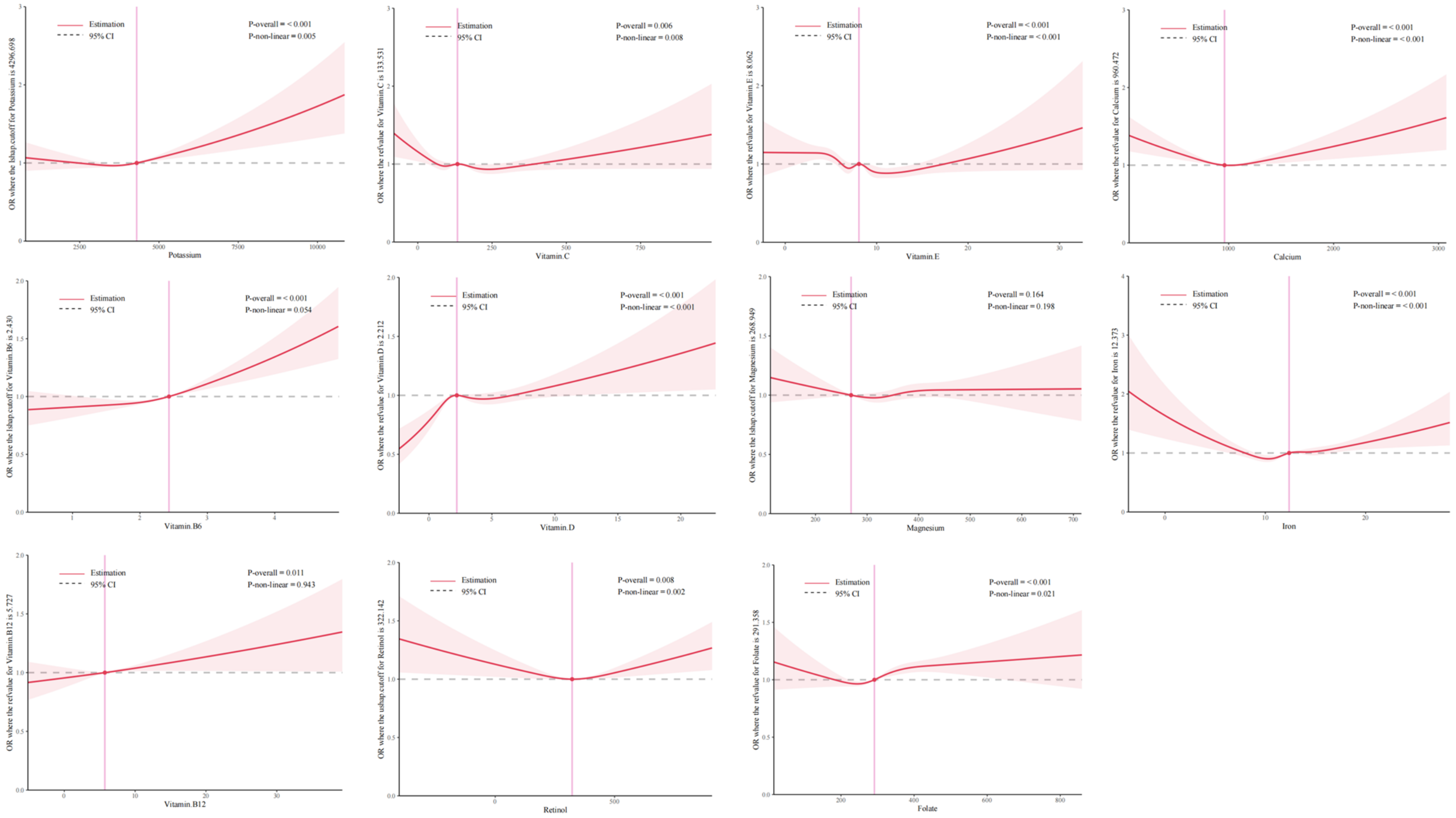

Supplementary Figure 10 Association between daily intake of micronutrients and risk of CVD. Intake of micronutrients were adjusted for total energy. Shaded areas represent 95% confidence intervals.

Supplementary Table 1 Characteristics of the study cohort, by incident case status for CAD in UK Biobank.[1]

Characteristics	Total	Non-CAD	CAD
Age, y, mean (SD)	58.50±7.85	58.31±7.85	62.52±6.70
Sex, n (%)			
Female	49,953(55.40)	48,556(56.41)	1,397(34.16)
Male	40,214(44.60)	37,521(43.59)	2,693(65.84)
Ethnicity, n (%)			
White	86,961(96.44)	83,012(96.44)	3,949(96.55)
Non-White	3,206(3.56)	3,065(3.56)	141(3.45)
Education years, n%			
≤7 years	4,683(5.19)	4,329(5.03)	354(8.66)
10-13 years	17,707(19.64)	16,880(19.61)	827(20.22)
15-20 years	67,777(75.17)	64,868(75.36)	2,909(71.12)
BMI categories, n%			
Underweight & Normal weight (<25)	36,689(40.69)	35,564(41.32)	1,125(27.51)
overweight (25 to <30)	36,708(40.71)	34,833(40.47)	1,875(45.84)
obese≥30*	16,770(18.60)	15,680(18.22)	1,090(26.65)
Smoking, n (%)			
Never	52,594(58.33)	50,542(58.72)	2,052(50.17)
Previous	31,183(34.58)	29,531(34.31)	1,652(40.39)
Current	6,390(7.09)	6,004(6.98)	386(9.44)
Alcohol intake, n (%)			
Nondrinker	21,360(23.69)	20,337(23.63)	1,023(25.01)
0 to <10 g/d	22,141(24.56)	21,167(24.59)	974(23.81)
10 to < 20 g/d	17,798(19.74)	17,075(19.84)	723(17.68)
≥20 g/d	28,868(32.02)	27,498(31.95)	1,370(33.50)
Physical activity, n (%)			

Low	22,298(24.73)	21,255(24.69)	1,043(25.50)
Moderate	47,361(52.53)	45,326(52.66)	2,035(49.76)
High	20,508(22.74)	19,496(22.65)	1,012(24.74)
SBP, n (%)			
<90 mmHg	23(0.03)	22(0.03)	1(0.02)
90 to < 140 mmHg	56,470(62.63)	54,591(63.42)	1,879(45.94)
≥140 mmHg	33,674(37.35)	31,464(36.55)	2,210(54.03)
Vitamin C, mg	151.64±90.32	151.8±90.12	148.7±94.39
Vitamin B6, mg	2.16±0.62	2.16±0.62	2.24±0.66
Vitamin B12, mg	6.50±3.71	6.49±3.70	6.67±3.79
Vitamin E, mg	9.43±4.00	9.44±3.99	9.25±4.19
Vitamin D, mg	2.87±2.24	2.87±2.24	2.95±2.23
Folate, ug	302.66±97.31	302.4±97.16	309.2±100.2
Retinol, ug	329.87±151.87	329.5±151.8	337.1±153.9
Potassium, mg	3778.45±1094.70	3775.2±1089.8	3845.8±1191.4
Magnesium, mg	350.97±95.21	350.8±95.00	355.5±99.43
Iron, mg	13.76±3.81	13.75±3.80	13.98±4.00
Calcium, mg	981.19±355.20	980.5±334.1	995.7±357.2

[1] Values are mean (standard deviation) or number (percentages) unless otherwise indicated. CAD, coronary artery disease; SD, standard deviation

Supplementary Table 2 Characteristics of the study cohort, by incident case status for stroke in UK Biobank.[1]

Characteristics	Total	Non-Stroke	Stroke
Age, y, mean (SD)	58.50±7.85	58.44±7.84	63.38±6.65
Sex, n (%)			
Female	49,953(55.40)	49,467(55.56)	486(42.93)
Male	40,214(44.60)	39,568(44.44)	646(57.07)
Ethnicity, n (%)			
White	86,961(96.44)	85,861(96.44)	1,100(97.17)
Non-White	3,206(3.56)	3,174(3.56)	32(2.83)
Education years, n%			
≤7 years	4,683(5.19)	4,595(5.16)	88(7.77)
10-13 years	17,707(19.64)	17,468(19.62)	239(21.11)
15-20 years	67,777(75.17)	66,972(75.22)	805(71.11)
BMI categories, n%			
Underweight & Normal weight (<25)	36,689(40.69)	36,296(40.77)	393(34.72)
overweight (25 to <30)	36,708(40.71)	36,233(40.70)	475(41.96)
obese≥30*	16,770(18.60)	16,506(18.54)	264(23.32)
Smoking, n (%)			
Never	52,594(58.33)	52,049(58.46)	545(48.14)
Previous	31,183(34.58)	30,693(34.47)	490(43.29)
Current	6,390(7.09)	6,293(7.07)	97(8.57)
Alcohol intake, n (%)			
Nondrinker	21,360(23.69)	21,104(23.70)	256(22.61)
0 to <10 g/d	22,141(24.56)	21,871(24.56)	270(23.85)
10 to < 20 g/d	17,798(19.74)	17,617(19.79)	181(15.99)
≥20 g/d	28,868(32.02)	28,443(31.95)	425(37.54)
Physical activity, n (%)			

Low	22,298(24.73)	22,030(24.74)	268(23.67)
Moderate	47,361(52.53)	46,777(52.54)	584(51.59)
High	20,508(22.74)	20.228(22.72)	280(24.73)
SBP, n (%)			
<90 mmHg	23(0.03)	23(0.03)	0(0.00)
90 to < 140 mmHg	56,470(62.63)	55,962(62.85)	508(44.88)
≥140 mmHg	33,674(37.35)	33,050(37.12)	624(55.12)
Vitamin C, mg	151.64±90.32	151.6±90.29	151.7±93.09
Vitamin B6, mg	2.16±0.62	2.16±0.62	2.23±0.67
Vitamin B12, mg	6.50±3.71	6.50±3.71	6.68±3.69
Vitamin E, mg	9.43±4.00	9.43±3.99	9.46±4.42
Vitamin D, mg	2.87±2.24	2.87±2.24	3.01±2.40
Folate, ug	302.66±97.31	302.6±97.22	310.4±104.2
Retinol, ug	329.87±151.87	329.7±151.8	341.6±160.7
Potassium, mg	3778.45±1094.70	3777.2±1092.6	3875.6±1248.0
Magnesium, mg	350.97±95.21	350.9±95.08	356.9±104.6
Iron, mg	13.76±3.81	13.76±3.81	14.12±3.97
Calcium, mg	981.19±355.20	981.0±334.7	994.9±373.6

[1] Values are mean (standard deviation) or number (percentages) unless otherwise indicated. SD, standard deviation
